# Supplementary figures and images for: Efficient repair of human genetic defect by CRISPR/Cas9-mediated interlocus gene conversion
Source: Life Med. 2023 Nov 13;2(5):lnad042. doi: 10.1093/lifemedi/lnad042 (PMC11749481; doi:10.1093/lifemedi/lnad042)

A

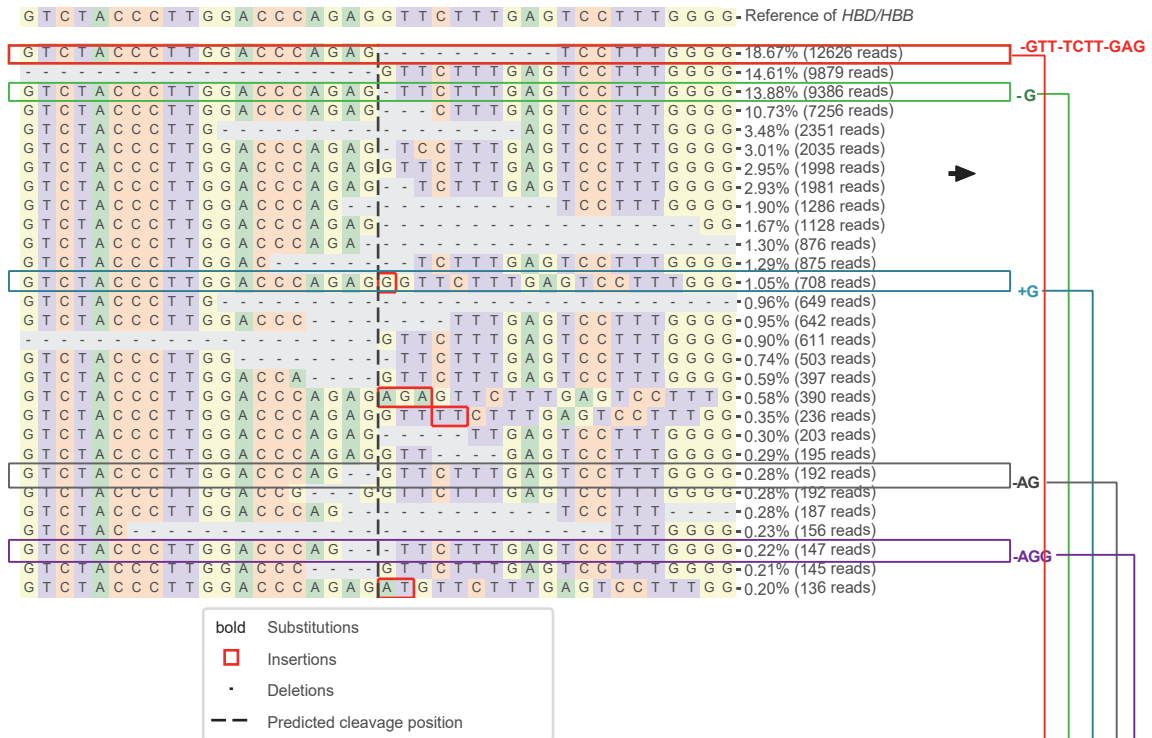

B

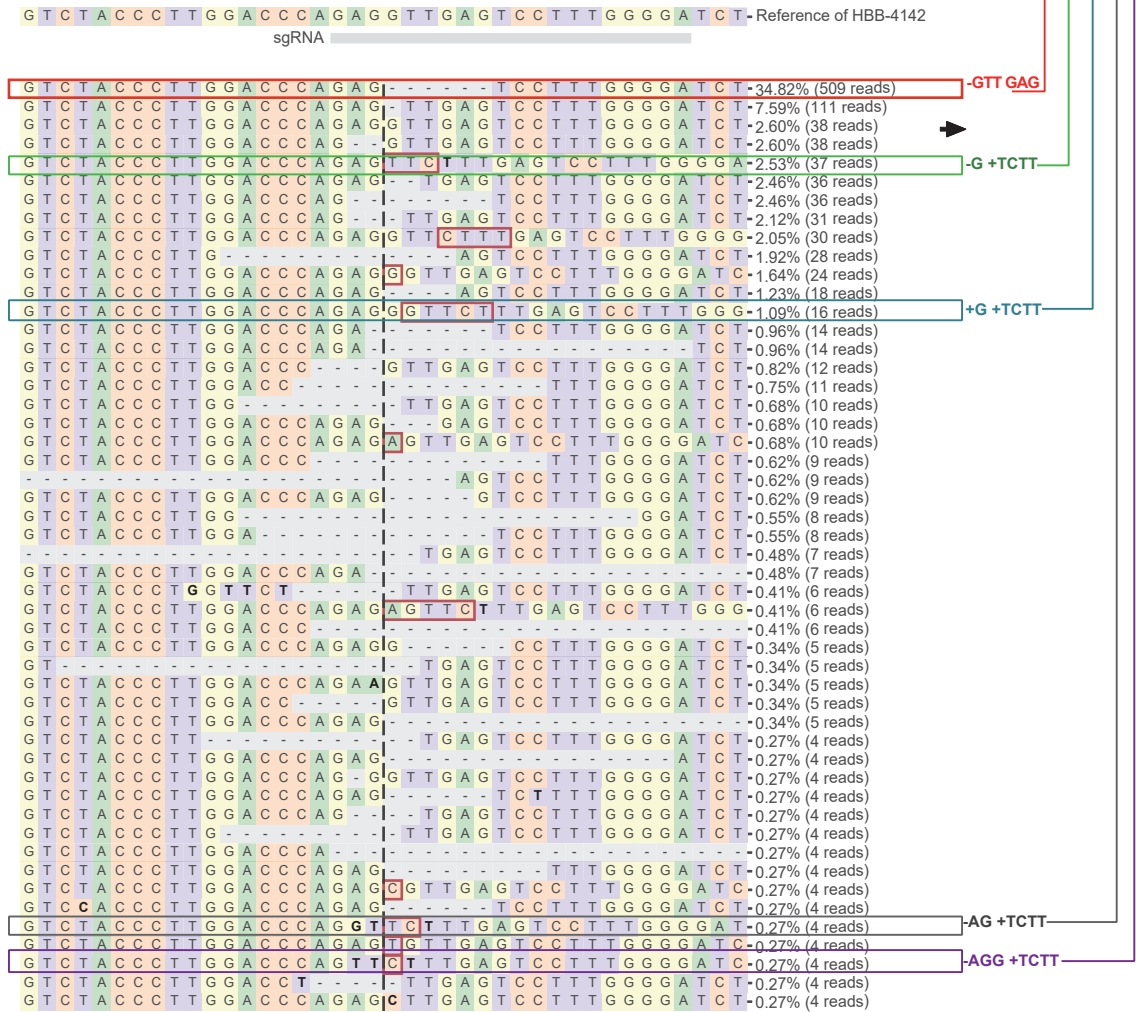

Supplement: lnad042_suppl_Supplementary_Figure_S1 [file lnad042_suppl_Supplementary_Figure_S1.pdf]

A

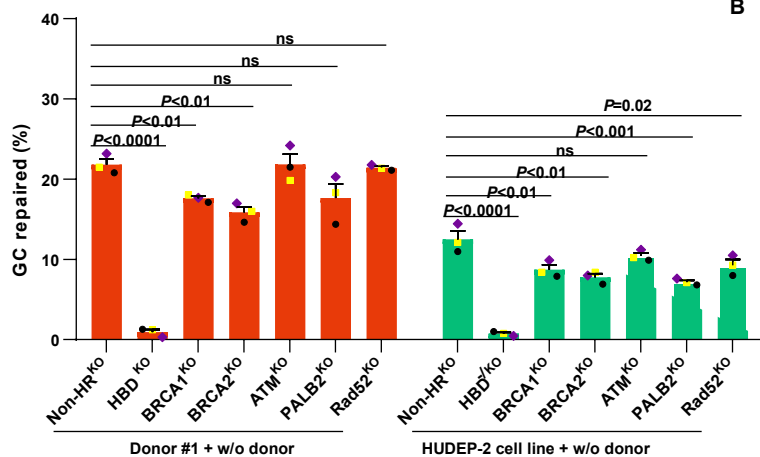

B

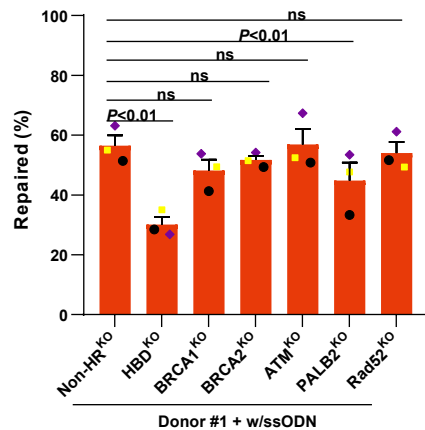

C

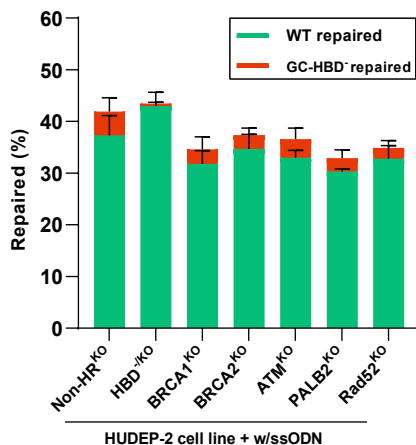

D

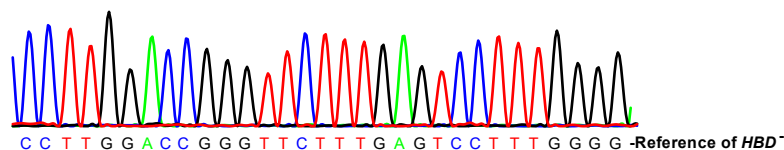

E

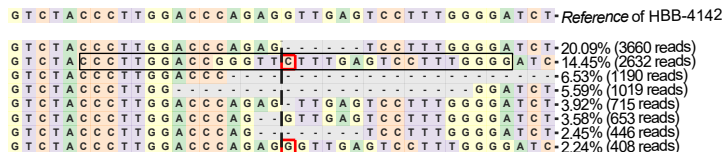

Supplement: lnad042_suppl_Supplementary_Figure_S3 [file lnad042_suppl_Supplementary_Figure_S3.pdf]

A

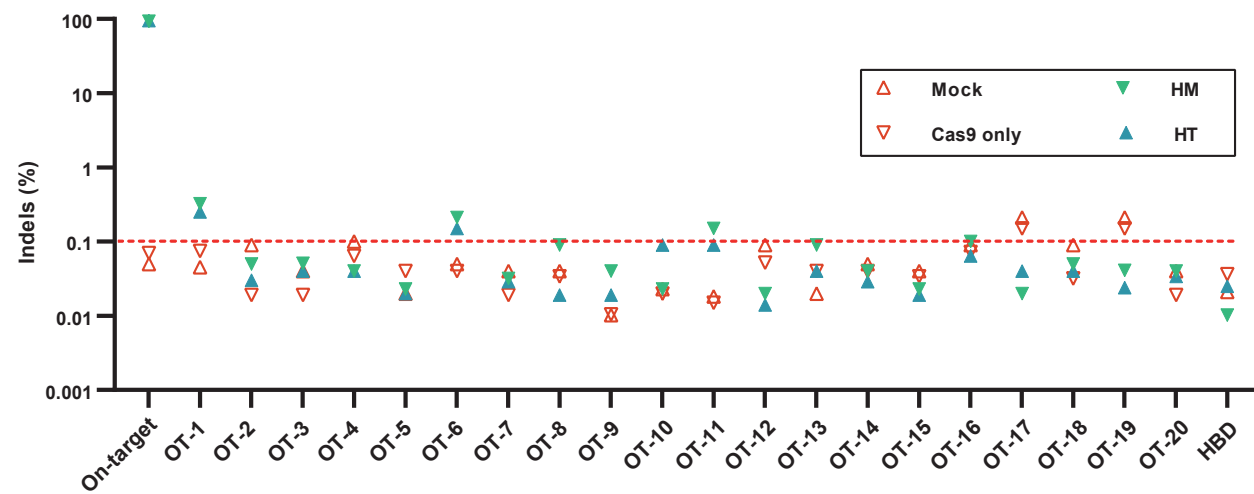

B

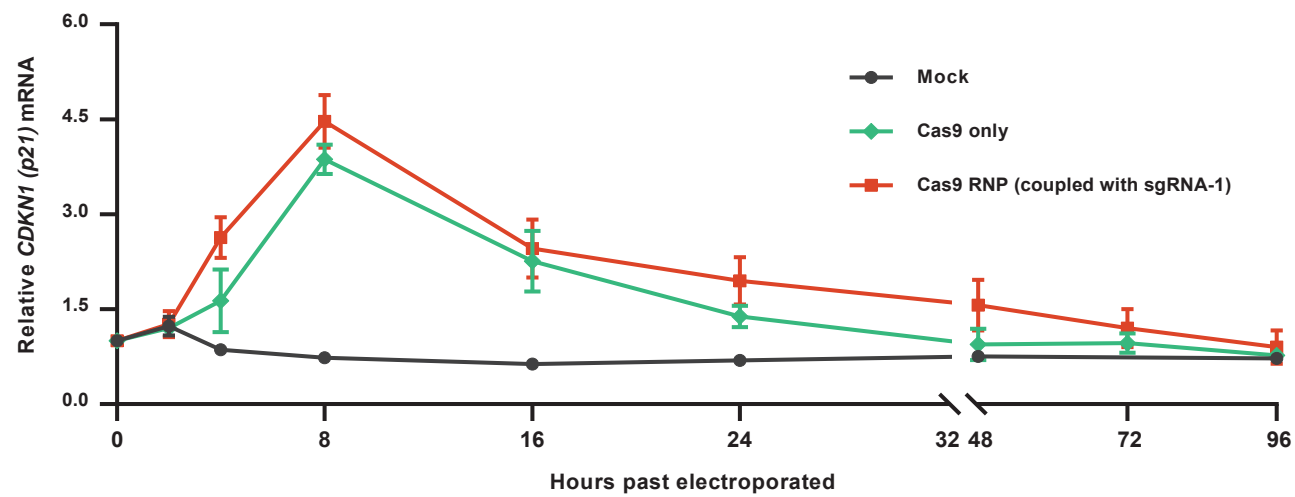

Supplement: lnad042_suppl_Supplementary_Figure_S4 [file lnad042_suppl_Supplementary_Figure_S4.pdf]

A

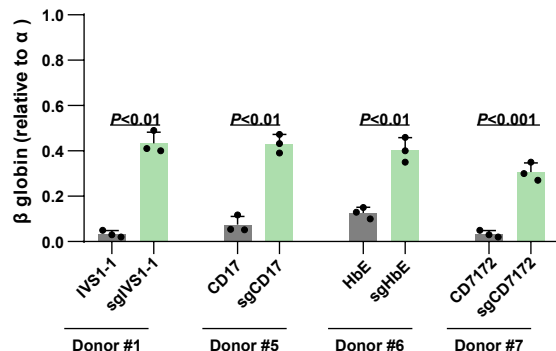

B

## CD7172 (+A) edited

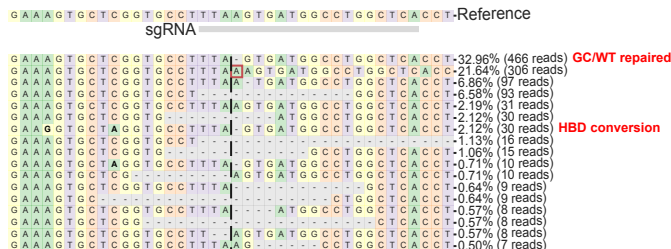

C

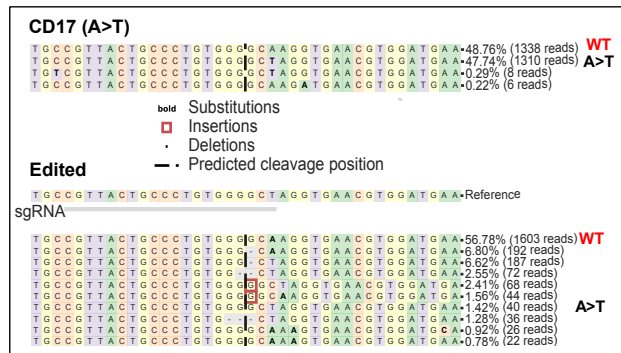

D

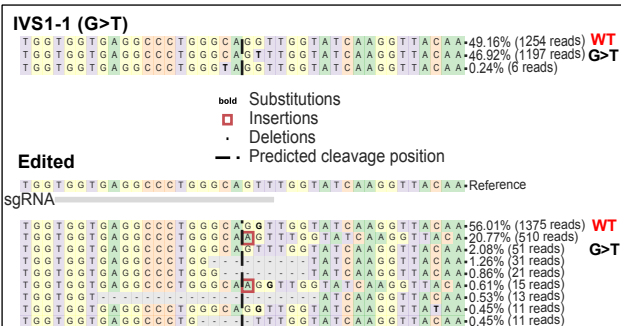

Supplement: lnad042_suppl_Supplementary_Figure_S5 [file lnad042_suppl_Supplementary_Figure_S5.pdf]
