# Supplementary material for: Efficient repair of human genetic defect by CRISPR/Cas9-mediated interlocus gene conversion
Source: Life Med. 2023 Nov 13;2(5):lnad042. doi: 10.1093/lifemedi/lnad042 (PMC11749481; doi:10.1093/lifemedi/lnad042)
Supplement: lnad042_suppl_Supplementary_Figure_S2 [file lnad042_suppl_Supplementary_Figure_S2.pdf]

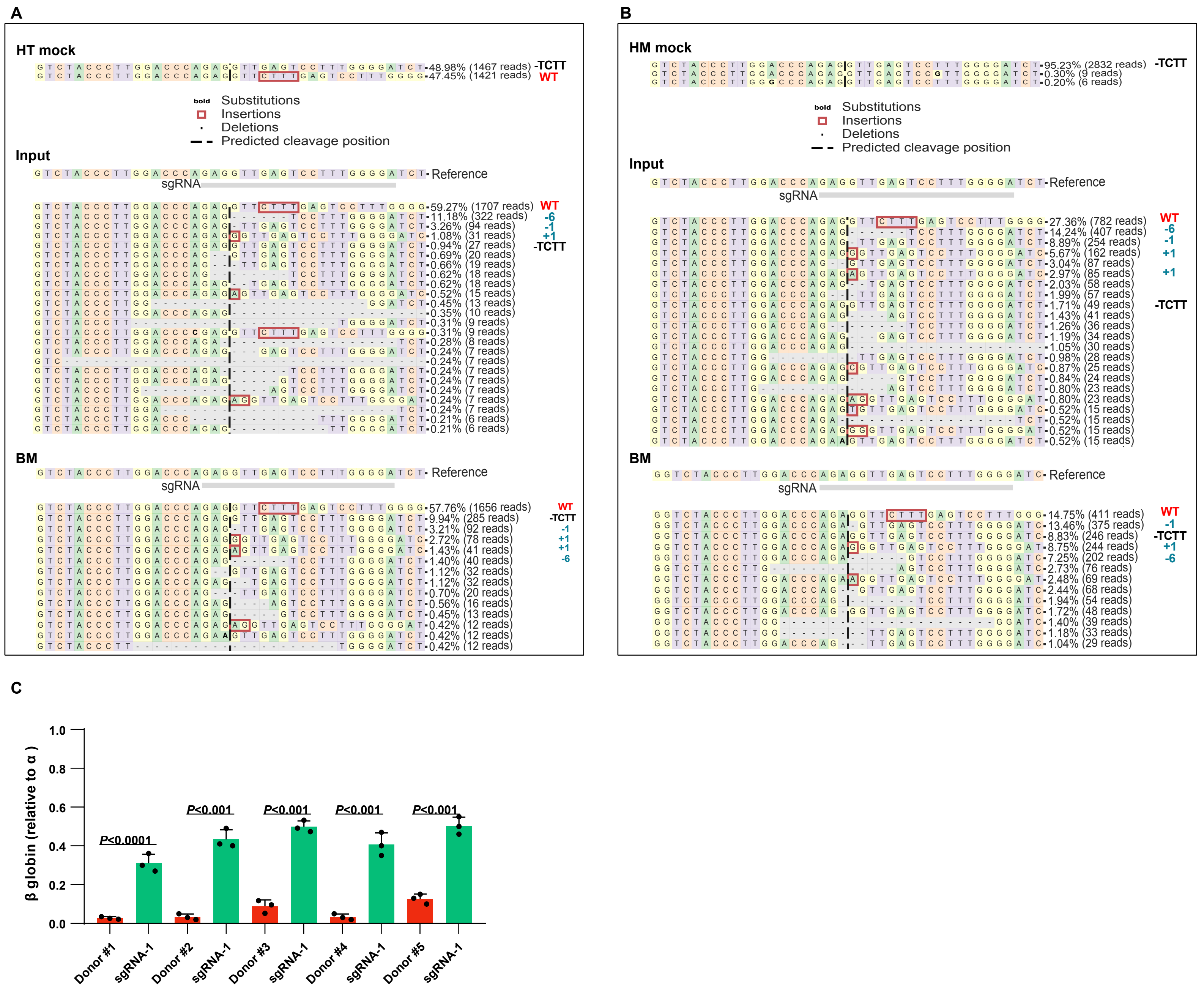
A
HT mock
-TCTT
WT
bold Substitutions
Insertions
Deletions
Predicted cleavage position
Input
Reference
sgRNA
BM
B
HM mock
C
β globin (relative to α)
1.0
0.8
0.6
0.4
0.2
0.0
P<0.0001
P<0.001
Donor #1
sgRNA-1
Donor #2
Donor #3
Donor #4
Donor #5
